# Balancing barriers: Family, career, and gender equality in radiation oncology and radiation research—An interdisciplinary prospective survey among the young workforce

**DOI:** 10.1007/s00066-025-02402-2

**Published:** 2025-05-27

**Authors:** Maike Trommer, Maximilian Grohmann, Alexander Fabian, Felix Ehret, Julia Hess, Michael Rückert, Johann Matschke, Sarah Stefanowicz, Alexander Rühle, Simone Ferdinandus, Ricarda Merten, Angela Besserer, Livia Schmidt, Elena Sperk, Alina Depardon, Florian Putz, Cordula Petersen, Marlen Haderlein, Annemarie Schröder, Thomas Weissmann, Lisa Deloch

**Affiliations:** 1 Department of Radiation Oncology, Faculty of Medicine and University Hospital Cologne, University of Cologne, Teutoburger Str. 5, 50678 Cologne, Germany; 2 Department of Radiation Oncology, Olivia Newton-John Cancer Wellness & Research Centre, Austin Health, Melbourne, VIC Australia; 3 Young DEGRO Trial Group, Berlin, Germany; 4 Center for Integrated Oncology Aachen Bonn Cologne Duesseldorf, Cologne, Germany; 5 Department of Radiotherapy and Radiation Oncology, University Medical Center Hamburg-Eppendorf, Martinistr. 52, 20246 Hamburg, Germany; 6 Department of Radiation Oncology, University Hospital Schleswig-Holstein, 24105 Kiel, Germany; 7 Department of Radiation Oncology, Charité—Universitätsmedizin Berlin, Corporate Member of Freie Universität Berlin and Humboldt-Universität zu Berlin, Berlin, Germany; 8 German Cancer Consortium (DKTK), partner site Berlin, a partnership between DKFZ and Charité—Universitätsmedizin Berlin, Berlin, Germany; 9 Research Unit Translational Metabolic Oncology, Institute for Diabetes and Cancer, Helmholtz Zentrum München Deutsches Forschungszentrum für Gesundheit und Umwelt (GmbH), Neuherberg, Germany; 10 Clinical Cooperation Group “Personalized Radiotherapy in Head and Neck Cancer”, Helmholtz Zentrum München Deutsches Forschungszentrum für Gesundheit und Umwelt (GmbH), Neuherberg, Germany; 11 Department of Radiation Oncology, University Hospital, LMU Munich, Munich, Germany; 12 Department of Radiation Oncology, Uniklinikum Erlangen, Friedrich-Alexander-Universität Erlangen-Nürnberg, Erlangen, Germany; 13 Translational Radiobiology, Department of Radiation Oncology, Uniklinikum Erlangen, Friedrich-Alexander-Universität Erlangen-Nürnberg, Erlangen, Germany; 14 Comprehensive Cancer Center Erlangen-EMN, Erlangen, Germany; 15 Institute of Cell Biology (Cancer Research), University Hospital Essen, University of Duisburg-Essen, 45147 Essen, Germany; 16 German Cancer Consortium (DKTK) partner site Essen a partnership between DKFZ and University Hospital, Essen, Germany; 17 Department of Radiation Oncology, Klinikum rechts der Isar, Technical University of Munich (TUM), Munich, Germany; 18 Department of Radiation Oncology, University of Freiburg—Medical Center, Freiburg, Germany; 19 Department of Radiation Oncology, University of Leipzig, Leipzig, Germany; 20 Department of Radiation Oncology, Ernst von Bergmann Hospital Potsdam, Potsdam, Germany; 21 Department of Radiation Oncology, University Hospital Düsseldorf, Düsseldorf, Germany; 22 Mannheim Cancer Center, Universitätsmedizin Mannheim, Medical Faculty Mannheim, Heidelberg University, Mannheim, Germany; 23 Department of Radiotherapy and Radiation Oncology, University Medical Center Rostock, Suedring 75, 18059 Rostock, Germany

**Keywords:** Parity, Gender, Radiation oncology, Young workforce, Survey

## Abstract

**Purpose:**

There is an urgent need to recruit and retain young professionals in radiation oncology and radiation research as the healthcare system faces major challenges. Our study investigated the experiences and needs of young professionals in this field, focusing on the impact of unpaid care work and gender-related issues.

**Methods:**

A web-based survey was created and distributed over a six-week period, featuring one general questionnaire along with three occupation-specific versions tailored for physicians, biologists, and medical physicists involved in radiation oncology and research.

**Results:**

Most participants with care responsibilities have temporary contracts, especially female physicians and biologists, while female medical physicists are more likely to hold permanent positions. Research is often conducted outside regular hours, with limited cover arrangements and part-time options varying by field. Key career risks include economic pressure, work-life balance, and uncertain contracts, with employees with care duties feeling less supported overall. In addition, men seem to be more involved in care work and thus face unique challenges, such as insufficient career support and fears of poor future perspective. The study emphasizes the need for strategies to address relevant issues, such as flexible working arrangements, better mentoring support, and clear substitution policies that can ensure that young professionals can balance caring responsibilities with work and career demands.

**Conclusion:**

Addressing these challenges is critical for sustaining a diverse and qualified workforce in radiation oncology and radiation research, ensuring excellence in patient care and scientific progress.

**Supplementary Information:**

The online version of this article (10.1007/s00066-025-02402-2) contains supplementary material, which is available to authorized users.

## Introduction

The present healthcare system is rapidly evolving, presenting multi-faceted challenges beyond clinical practice and socioeconomic dimensions. In recent years, we have seen an increasing awareness of the potential shortage of professionals ensuring patient care despite the expected rise in patient numbers, as well as the need to secure state-of-the-art research in radiation oncology and radiation research [1]. As reported by various studies within the last years [2–5], there is thus an urgent need to assess the wishes and needs of the young workforce to secure sufficient numbers of qualified professionals in radiation oncology and radiation research. While the majority of studies have focused on the medical workforce, our previous publication examined the needs and wishes of young professionals from all subspecialties involved in radiation oncology and radiation research [2]. While some subspecialty-specific needs were identified, we also found that many challenges are perceived across disciplines [2].

One factor that has not been sufficiently considered, is whether care work can have a significant impact on the identified challenges of the younger workforce. Terms such as gender equity, gender pay gap, and the compatibility of family and career are often perceived as major issues in the general population [6–10]. In the survey performed by Weissmann et al. [2], economic pressure, work-life balance, and uncertain contract terms were the most named issues that arise in long-term career planning in radiation oncology.

The evolving healthcare landscape, particularly within the field of radiation oncology, presents a multifaceted challenge that extends beyond the fields of clinical practice and into the broader socio-economic and cultural dimensions [11, 12].

The increasing number of female medical students in Germany constituted for 64.3% of all medical students in 2022 [13]. There seem to be no gender-specific differences in the interest to pursue a training in radiation oncology in Germany [14]. After starting their career as radiation oncologists, women tend to drop out disproportionately with each career step, a phenomenon known as the “leaky pipeline” [15]. This phenomenon might be exacerbated by the significant yet often underappreciated role of “unpaid care work” (UCW) [16]. The double burden of both UCW and professional obligations could disproportionately affect women [17], raising concerns about gender equity and the barriers to combining a functioning family life with professional duties [18]. It is likely that this is also true for the other professional fields within radiation oncology. Women also did carry the biggest burden in UCW during the COVID-19 pandemic, as mothers were more likely to care for children and the household. Nevertheless, while still staying below the percentage of UCW of women, a survey did indicate a slight increase in UCW also in men during the pandemic in some countries [19]. However, despite this increase in participation, in general, men still spend significantly less time in UCW than women, and more time in paid work [20, 21]. Accordingly, women also felt significantly more burdened during the pandemic, according to a survey carried out within the German radiation oncology workforce [22]. However, today the gender gap in care work has returned to pre-pandemic levels, with Germany still having one of the highest gender differences in international comparison, according to a publication by the German Institute for Economic Research (*Deutsches Institut für Wirtschaftsforschung*) [23]. Engaging men in UCW not only supports gender equality but also has positive effects on family dynamics and children’s development. Increased male involvement can thus lead to better outcomes for children and contribute to more balanced partnerships [19]. This can ultimately lead to increased satisfaction of the younger workforce, also in Germany. Unfortunately, in Germany, income limits for parental allowance recently has decreased for couples from € 300,000 to € 200,000, and for single parents, from € 250,000 to € 150,000. Subsequently, for births from April 1, 2025, this threshold will be further reduced to € 175,000 for both, couples and single parents. In addition, there were also changes regarding the partner months: While it previously did not matter whether these months were taken simultaneously or consecutively, now, one of the partner months must be taken simultaneously within the first twelve months of a child’s life. These adjustments aimed to allocate resources more effectively and promote a balanced distribution of parental responsibilities. By tightening eligibility criteria, the government seeks to encourage both parents to participate more equally in childcare, moving away from traditional models where mothers are primary caregivers and fathers are primary earners. However, these changes impact higher-earning families (such as parents in the medical workforce) who previously qualified for parental allowance, potentially influencing their decisions regarding parental leave and workforce participation. Especially for women, it may be more attractive to work and earn less to receive parental allowance during the time with UCW and thus without relevant income.

It is therefore necessary to identify and implement strategies that promote gender equity, recognize and appreciate care work, and create a supportive working environment in the field of radiation oncology and radiation research. This includes rethinking funding priorities and a flexible working environment, improving the appreciation of care work, and fostering a more inclusive and supportive professional culture. To achieve this, a fundamental understanding of the needs and wishes of professionals of all gender working in radiation oncology and radiation research needs to be identified. Furthermore, a better understanding of how and whether care work changes these requirements also needs to be identified.

## Materials and Methods

### Survey

Within the framework of the young DEGRO (*Deutsche Gesellschaft für Radioonkologie*, German Society for Radiation Oncology) Trial Group, young DeGBS (*Deutsche Gesellschaft für Biologische Strahlenforschung*, German Society for Biological Radiation Research) and young Medical Physics (*Deutsche Gesellschaft für Medizinphysik* (DGMP), German Society for Medical Physics), a web-based questionnaire with one general as well as three occupational-specific questionnaires for physicians, biologists and medical physicists working in radiation oncology and radiation research was developed. The survey contained 80 questions in total, each occupational group had to answer 13 general questions independent of their profession (part A) and 25, 22 or 20 occupation-related questions (part B), respectively. Only participants that have fully completed the questionnaire were included in the analysis. Further information on the survey are provided in Weissmann et al. [2]. Due to the very low number of participants involved in care work that chose “diverse” or “I’d rather not say” in the survey, we decided to exclude these participants to ensure answers cannot be traced back to the participants.

### Statistics

Statistical analysis was carried out using GraphPad Prism© (GraphPad Software, LLC, Version 9.5.1, Boston, Massachusetts USA). Descriptive Statistics were used to visualize answers given by the participants.

### Ethics

The survey was conducted in accordance with the ethical standards of the Helsinki Declaration of 1964 and its subsequent amendments, and/or comparable standards. The questionnaire was provided on the basis of and in accordance with the regulations of DEGRO’s data protection conditions with Enuvo Inc., Switzerland. In accordance with the ethics Committee in Erlangen as well as in line with the recommendations of the joint ethics committee of the Bavarian universities (GEHBa), no additional approval was necessary for the conducted survey. Further steps regarding the methodology have been previously described [2].

## Results

### Participant characteristics

**Fig. 1:**
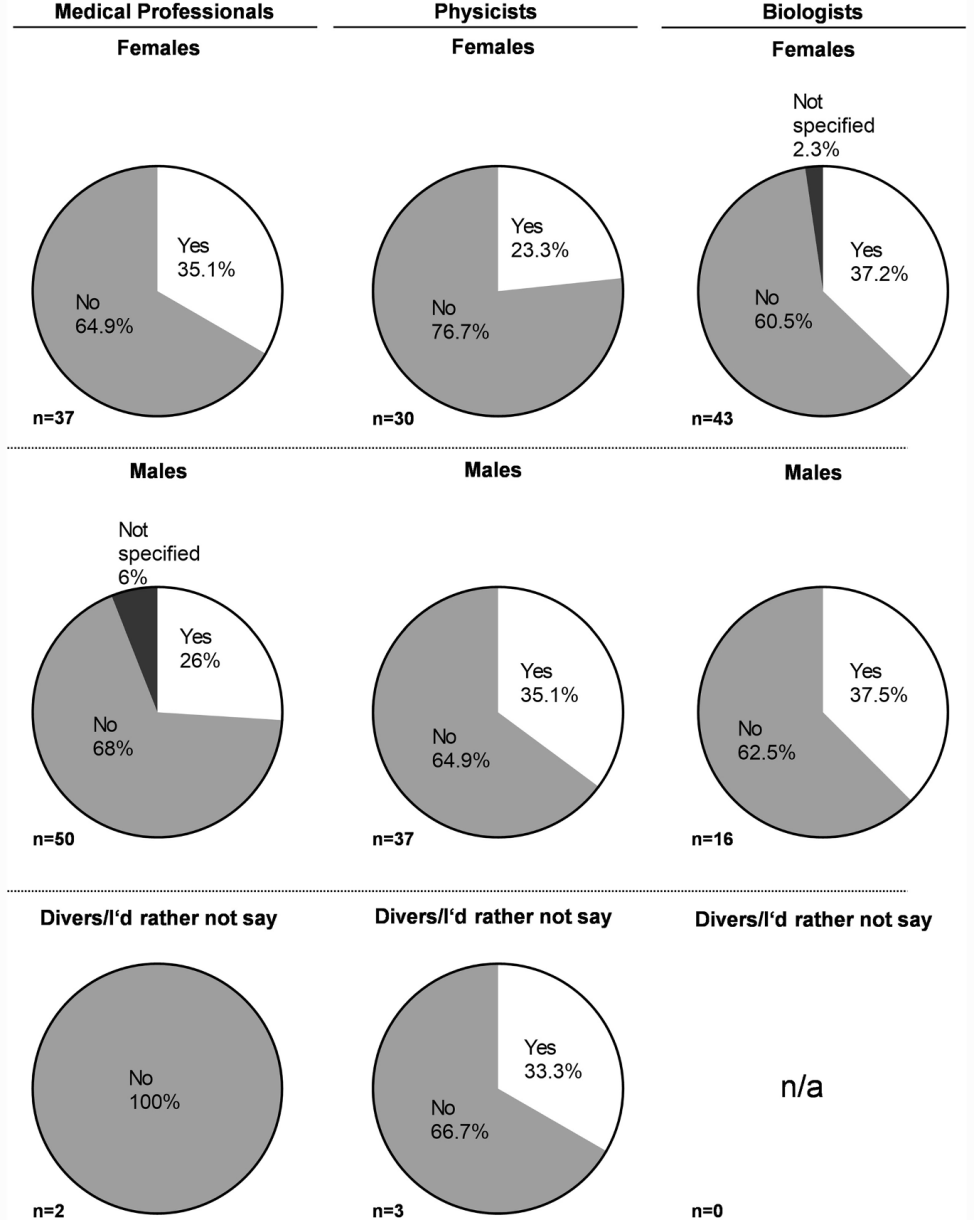
Overview of gender distribution and the answers given to the question: “Do you have children or relatives to care for?” The gender distribution of all participants was according to their subspeciality, and the distribution of participants involved in care work was divided according to gender. Numbers are given in %, with total numbers added to the bottom of each graph

A total of 218 people took part in the survey, 89 radiation oncologists, 59 radiation biologists and 70 medical physicists [2] and Supplementary Fig. 1. 30% (*n* = 27) of radiation oncologists, 37.3% (*n* = 22) of radiation biologists, and 30% (*n* = 21) of medical physicists indicated to either have children or relatives to care for, Fig. 1.

While the general age distribution in all participants ranged from below 20 to above 40 years of age, age distribution of participants with care work ranged from 26 to > 40 years, (Supplementary Fig. 1).

### Workload, overtime hours and employment contracts

**Fig. 2:**
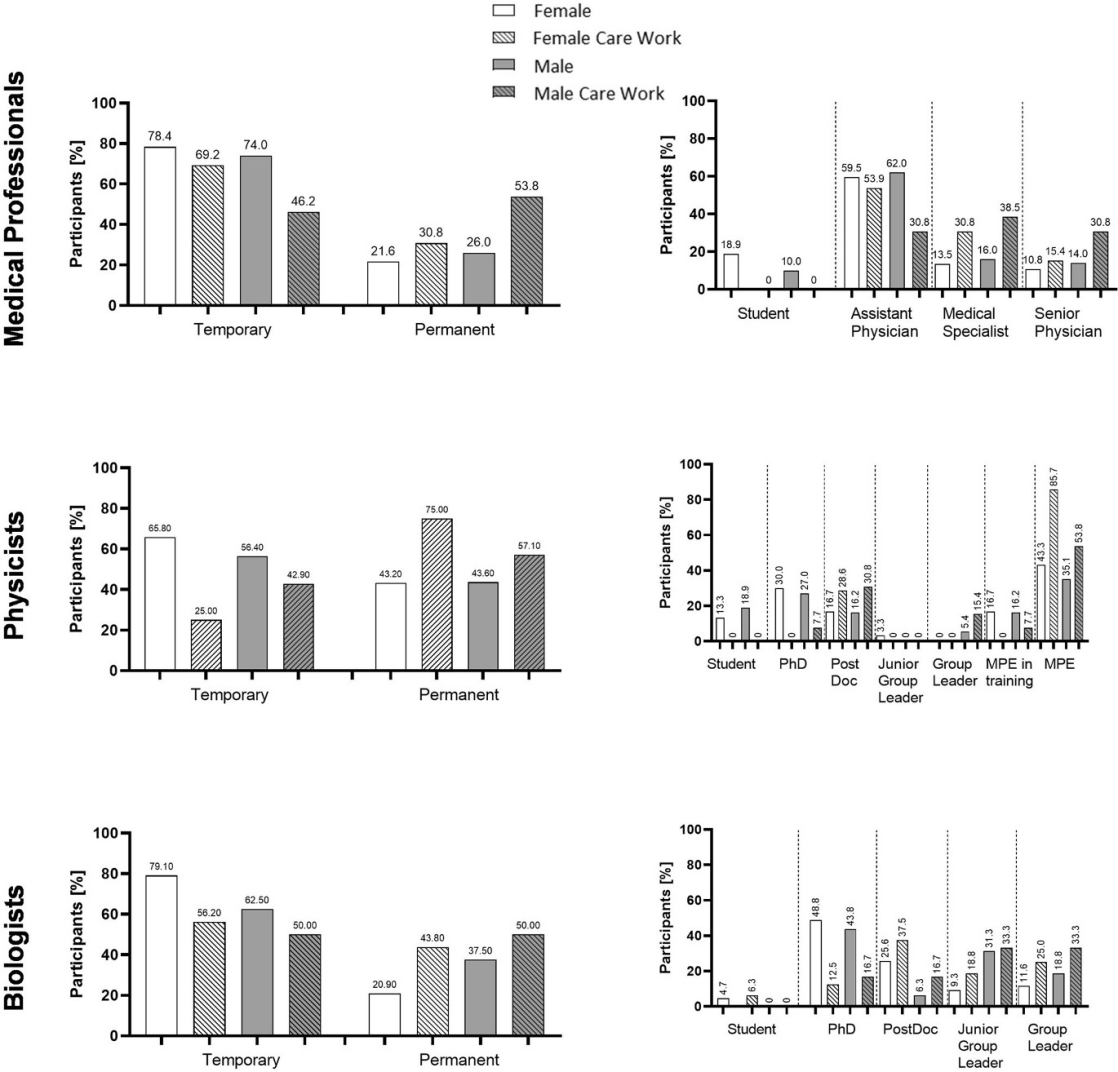
Temporary and permanent working contracts as well as career level of participants within the subspecialties. Overview of the contract situation and career level in all participants and those involved in care work distributed by gender. Numbers are given in %. For medical physicists, multiple selections were possible, e.g., a participant could be a group leader while also holding a medical physicist expert (*MPE*) title

Of all participants with care responsibilities, 37 stated to have a permanent employment contract. Overall, 17% of all participants and 43.5% of participants with care responsibilities indicated to have permanent positions. For distribution among the three disciplines regarding temporary or permanent employment contracts, see Fig. 2. For participants involved in care work, more female physicians (69.2% temporary vs. 30.8% permanent) and female biologists (56.2% temporary vs. 43.8% permanent) have temporary working contracts, while more female medical physicists are employed permanently (25% temporary vs. 75% permanent). Male physicians and medical physicists involved in care work more often have a permanent working contract in contrast to the total number of participating physicians and medical physicists while male biologists involved in care work are equally temporary or permanently employed (Fig. 2).

Regarding career levels, for medical professionals, most of participating males and females were holding assistant physician positions (m: 62%, f: 59.5%). Participating physicians involved in care work were mostly at medical specialist level for males (38.5%) and assistant physician level for females (53.39%). For medical physicists, most male and female participants were holding a medical physicist expert (MPE) title (m: 35.1%, f: 43.3%). When only looking at medical physicists involved in care work, that was still the case, while overall a larger percentage of those involved in care work were holding their MPE, in comparison to overall participants (m: 53.8%, f: 85.7%). However, when looking into career levels, most participating medical physicists were at PhD level (m: 27%, f: 30%) whereas most medical physicists involved in care work were at PostDoc level (m: 30.8%, f: 28.6%). The largest group of participating biologists was at PhD level (m: 43.8%, f: 48.8%). However, for biologists involved in care work, most male participants were equally distributed onto Junior group Leader and Group Leader level (33.3% each). In contrast, females involved in care work were mainly at PostDoc level (37.5%) with only 18.8% holding a Junior Group Leader Position and 25% a Group Leader Position.

**Fig. 3:**
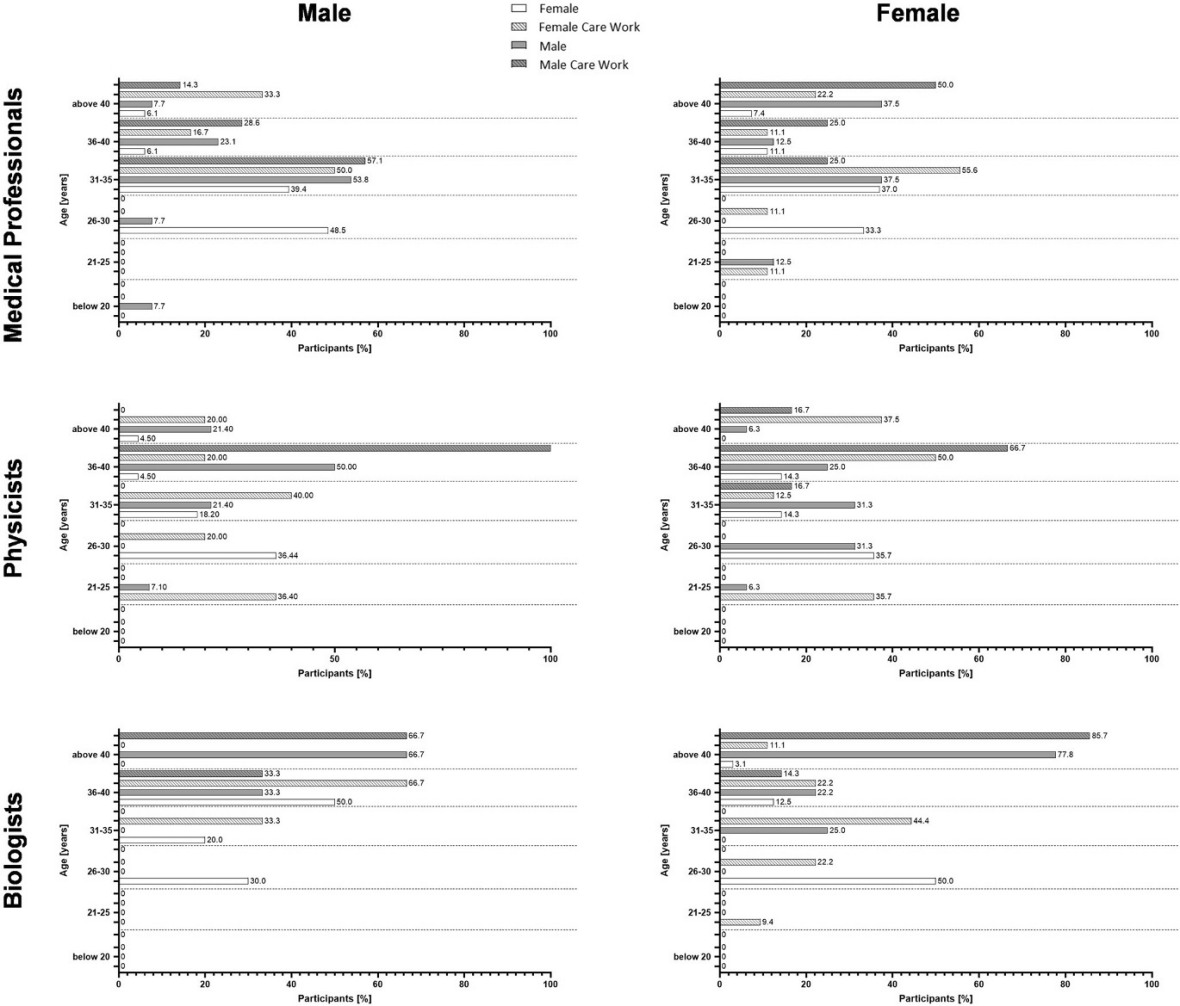
Contractual situation divided according to age, gender and involvement of care work. Overview of the contract situation in all participants and those involved in care work distributed by gender and age. Numbers are given in %

We also looked into age distribution subject to contractual situations, as depicted in Fig. 3. Within the physician group, male physicians aged 26–30 held the most temporary contracts (48.5%), while those aged 31–35 were the largest group with permanent positions (53.8%). For female physicians, temporary contracts were most common in the 31–35 age range (37%), whereas permanent contracts were evenly distributed between 31–35 and 40+ (both 37.5%). Among physicians with care work, male participants in the 31–35 range dominated both temporary (50%) and permanent positions (57.1%), while for females, the most temporary contracts were in the 31–35 age range (55.6%), with permanent contracts most common above 40 (50%). Overall, 26% of male and 21.6% of female physicians held permanent positions.

Among male medical physicists, temporary contracts were most common in younger age groups (21–25 and 26–30; both: 36.4%), while permanent contracts peaked at 36–40 (50%). Care work shifted these numbers slightly, with most temporary contracts at 31–35 (40%) and permanent contracts concentrated at 36–40 (100%, *n* = 1). Female medical physicists with temporary contracts were mostly aged 21–30 (both 35.7%), while permanent contracts increased with age, especially at 36–40 (66.7%). Medical physicists are the only subspeciality with more female than male permanent position holders. (53.3% vs. 37.8%).

Biologists had the latest entry into permanent contracts, with 6 out of 16 male (37.5%) and 9 out of 43 female biologists (20.9%) holding a permanent position. Male biologists transitioned to permanent contracts only at 36–40 (33.3%) and beyond (66.7%), while temporary contracts were most common in this age range of 36 to 40 (50%). Female biologists holding temporary contracts were predominantly aged 26–30 (50%), while the largest age group of those holding a permanent position was above 40 years of age (77.8%). Care work shifted these patterns slightly, with older participants more likely to hold permanent positions (males: 36–40 (66.7%); females: 40 and older (85.7%), Fig. 3).

Regarding the workload, we compared total participants with participants with care responsibilities. An in depth analysis of workload in research and clinic of all participants, was recently published by Weissmann et al. [2]. Work hours of employers with care work did not alter too much from work hours of all employees, Supplementary Fig. 2.

Most of the participating physicians and medical physicists indicated to be also working in research next to the clinic (data not shown): Only 4% of all male participating physicians indicated to not be carrying out any research (2 out of 50), while all male physicians involved in care work (*n* = 13) indicated to also carrying out research. For female physicians, amongst all participants 27% said they do not carry out research (10 out of 37) while for female physicians in care work numbers increased slightly to 38.5% (5 out of 13). Within the medical physicist cohort, 13.5% of participating males said they are not involved in research (5 out of 37) and 33.3% of female medical physicists (10 out of 30) also indicated to not be involved in research. For medical physicists involved in care work, numbers of those not carrying out research increased for both sexes: males 15.4% (2 out of 13) and 42.9% (3 out of 7) females.

We further asked, if potential research work would be performed within the regular working hours (Supplementary Fig. 3). Most physicians performing research did this outside their normal working contract time (m: 74.5%, f: 59.3%) or mostly out of their regular working time (m: 17%, f: 18.5%). Only a small group carried out research during their normal working hours (m: 4.3%, f: 7.4%). Medical physicists performing research without care work did this, most of the time, within working hours. For medical physicists involved in care work, while numbers of male medical physicists involved in care work said they do not carry out research during normal working hours decreased (9.1% instead of 13.3%), numbers in female medical physicists involved in care work that said they do not carry out research during normal working hours increased (50% of those involved in care work vs. 15.8% all female medical physicists).

In contrast, most of the biologists performing research without care work or with care work did this within their normal working hours. No biologists involved in care work carry out research outside of regular working hours, however, 6.3% of females have indicated to have other arrangements.

### Cover arrangements

**Fig. 4:**
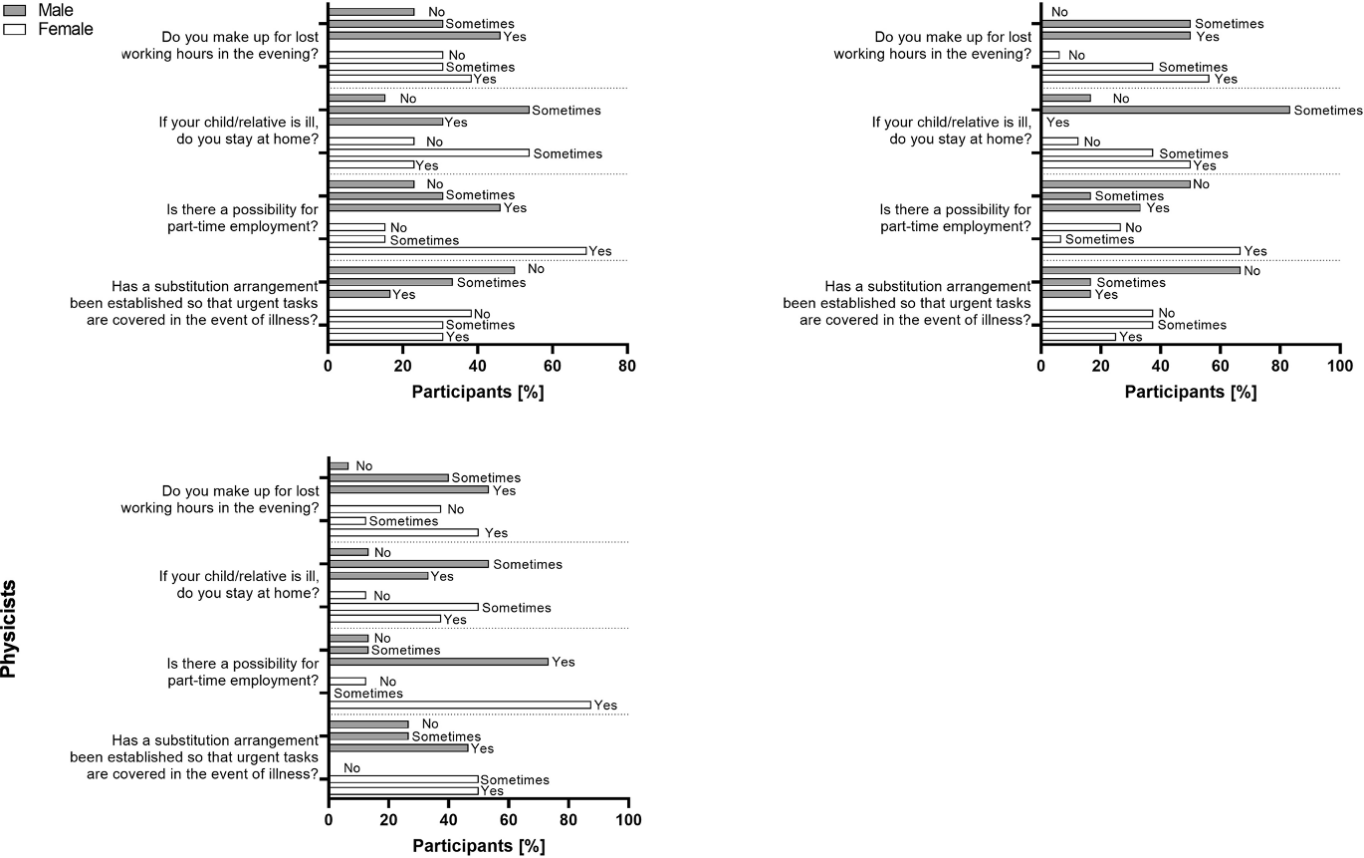
Arrangements for employees being involved in care work. Overview of the arrangements for employees involved in care work, separately shown for each subspeciality and gender. Numbers are given in %

Of all participants with care responsibilities, a total of 37% stated that there were no cover arrangements for urgent tasks in the event of an unscheduled (e.g., carer’s) leave. They also stated that 48% would make up for their work and the hours they had missed during the day in the evening. That affected physicians in 46.2% (m) and 38.5% (f) of the cases, biologists in 50% (m) and 56.3% (f) of the cases and medical physicists in 53.3% (m) and 50% (f) of the cases (Fig. 4). In case of unexpected sickness, females tend to stay home more often than males in the biologist and medical physicist workgroup, but not in the physician group, while most employees only stayed at home “sometimes”. In many cases, participants report that part-time employment is possible, whereas the highest numbers are found in the physicist group (physicians: 46.2% (m), 69.2% (f); biologists: 33.3% (m), 66.7% (f); physicists: 73.3% (m), 87.5% (f)). Among the three groups, part-time employment is least possible in the male biologist group, followed by female biologists (“No, there is no possibility for part-time employment”; physicians: 23.1% (m), 15.4% (f); biologists: 50% (m), 26.7% (f); physicists: 13.3% (m), 12.5% (f)).

### Biggest risk factors for career termination

Key risk factors preventing long-term careers in radiation oncology and research differ by subspecialty, gender, and care responsibilities.

For physicians, in the total workforce, the major concern for male and female employees is economic pressure (m: 60%, f: 48.6%). The risk factors where male and females differ most are a lack of equality (m: 6%, f: 32.4%), the economic pressure, a lack of future educational possibilities (m: 8%, f: 18.9%), as well as the lack of compatibility of career and family (m: 30%, f: 40.5%). In physicians with care work, economic pressure remains the major risk factor (m: 53.8%, f: 53.8%), however, gender differences emerge in future perspectives: Here, the biggest differences are found in a lack of future perspectives (m: 53.8%, f: 23.1%), lack of equality (m: 15.4%, f: 38.5%), the lack of future educational possibilities (m: 0%, f: 23.1%) and work/life balance (m: 46.2%, f: 38.5%).

In the medical physicist group, the biggest concerns in male and female participants differ: here, males identify economic pressure as their biggest concern (m: 35.1%, f: 20%), and females chose work/life balance (m: 27%, f: 36.7%). The biggest differences in genders were found in the lack of equality (m: 5.4%, f: 30%), lack of compatibility of family and career (m: 16.2%, f: 33.3%), and lack of future perspectives (m: 32.4%, f: 16.7%). Major concerns also shifted in those involved in care work: Here, the biggest concern for males was equally distributed towards “other” (e.g.: “lack of support from seniors”, “difficulty combining research and clinical work”, “lack of future perspectives”; m: 30.8%, f: 28.6%), economic pressure (m: 30.8%, f: 14.3%), and lack of future perspectives (m: 30.8%, f: 14.3%). For females the biggest factor is the lack of compatibility of family and career (m: 23.1%, f: 42.9%). The biggest differences between male and female participants involved in care work are the compatibility of family and career, lack of future perspective and economic pressure, as well as the lack of future education possibilities (m: 15.4%, f: 0%).

**Fig. 5:**
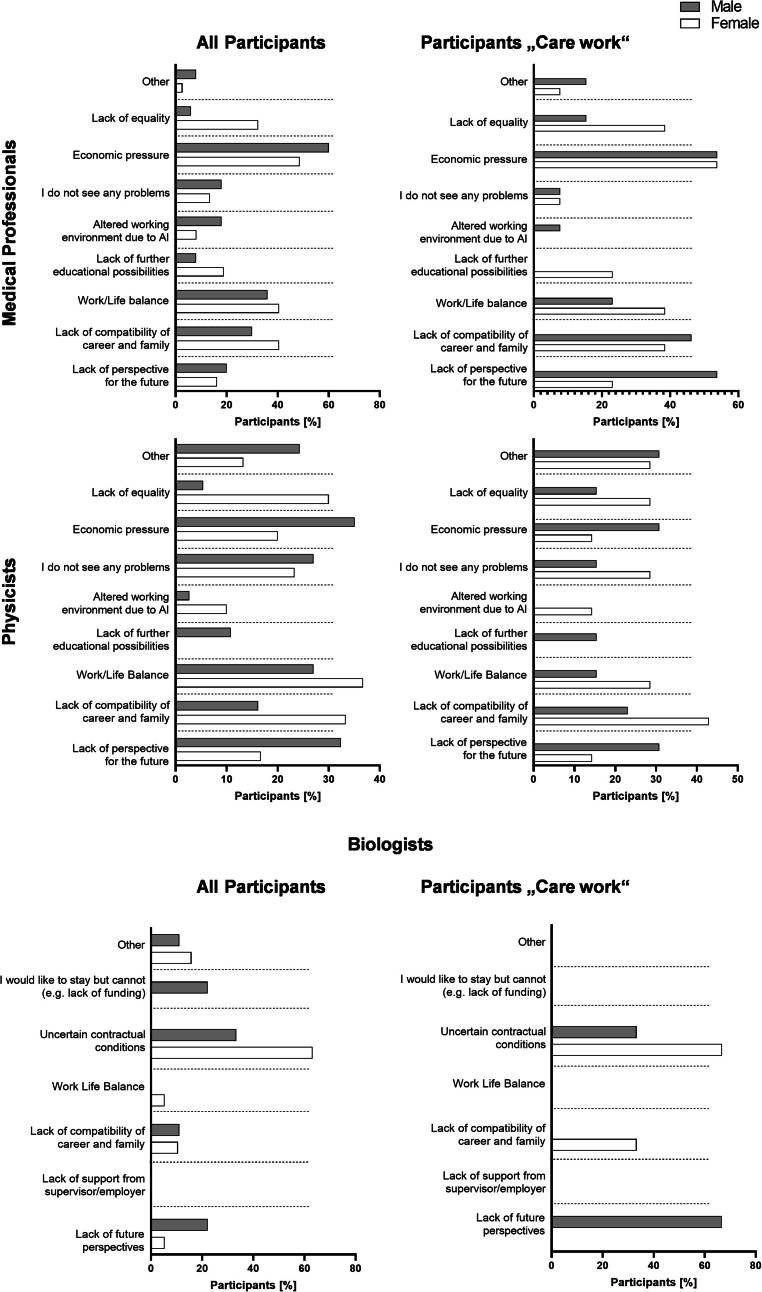
Major risk factors jeopardizing long-term career planning in the young workforce overall and those with care work. Participants were asked to identify their major concerns that would prevent them from pursuing a long-term career in radiation oncology and radiation science. Numbers are given in %

As biologists are not involved in clinical routine the same way physicians and physicists are, different questions were asked in this subspecialty. Among all participants, uncertain contract conditions are the leading concern for both genders (m: 33.3%, f: 63.2%), which was also the risk factor with the biggest difference between male and female perception. This was followed by a career termination due to a lack of funding (m: 22.2%, f: 0%) and the lack of future perspectives (m: 22.2%, f: 5.3%). In biologists involved in care work, perception shifts, and while females still identify uncertain contract situations as their major point of concern (m: 33.3%, f: 66.8%), males now choose the lack of future perspective as their major risk factors for potentially terminating a career in radiation research (m: 66.7%, f: 0%). Biggest differences between male and female biologists involved in care work are the lack of future perspective, uncertain contract situations, and the lack of compatibility of family and career, especially for females (m: 0%, f: 33.3%), Fig. 5.

### Support by employers and future perspective

In the medical field, regarding all participants, male physicians feel better supported than female physicians (m: 54%, f: 48.6%). Regarding physicians with care responsibilities, the feeling of support reverses in female and male participants, and male physicians feel less supported than female physicians (m: 38.5%, f: 53.8%).

Regarding biologists with or without care responsibilities, male participants feel better supported than female participants (m: 62.5%, f: 41.9%), and 12.5% of male and 34.9% of female biologists have not discussed career goals with their employer. In biologists with care work, there were no participants who haven’t discussed career goals, and 66.7% of males and 43.8% of females feel sufficiently supported by their employer.

Female physicists, in general, feel more supported than male physicists (m: 43.2%, f: 53.3%); physicists with care work show a similar trend, with 57.1% of females and 38.5% of males feeling sufficiently supported by their employer. When looking at the proportion of participants with care work versus all participants, 43.1% of participants with care work feel not supported (physicians: 46.2%, biologists 40.9%, physicists 42.9%), while only 31.4% of all participants feel not supported (physicians: 31.5%, biologists 48.3%, physicists: 24.3%).

**Fig. 6:**
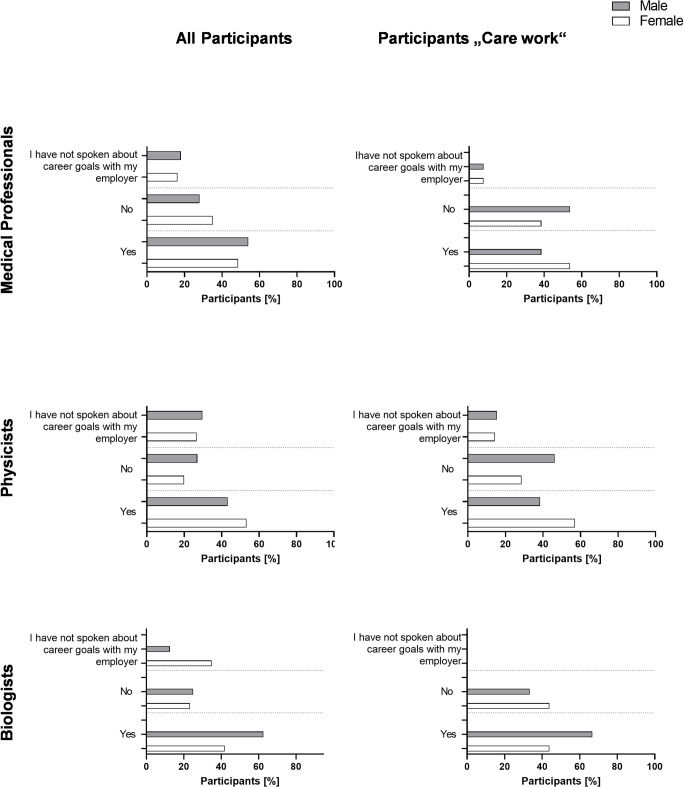
Does your employer provide sufficient support regarding your career goals? Participants were asked if they felt like they received a sufficient amount of support from their employer. All answers are given in [%] either for all participants or those with care work

Answers regarding support in career goals by all participants and participants with care work are demonstrated in Fig. 6.

## Discussion

This study aimed to examine the current situation of young professionals with care responsibilities across the subspecialties within radiation oncology and radiation research (physicians, biologists, medical physicists) in Germany, while also providing an insight into gender-specific differences and needs.

Several studies have highlighted concerns about workforce shortages in radiation oncology across Europe: Datta et al. (2014) projected disparities in radiotherapy staffing and infrastructure up to 2020, while Lievens et al. (2020) emphasized regional variations and insufficient staffing levels that could impact optimal radiotherapy delivery [24, 25]. The increasing demographic changes as well as the anticipated retirement of a high proportion of qualified personnel underlines the urgent need for strategies to attract and retain young professionals in radiation oncology [26, 27].

Within our study, the proportions of participants involved in care work were around 1/3 for all groups with biologists showing a marginally higher rate (see Fig. 1), which could be potentially due to the higher rates in female participants in biology (Supplementary Fig. 1). In general, more women than men are involved in UCW, which was especially shown during the COVID-19 pandemic [22, 28, 29], as evidenced by the International Labor Organization. The study revealed that 708 million women and 40 million men worldwide are not economically active due to their unpaid care responsibilities, a figure consistent with the situation in Germany [23, 30]. The gap in UCW during the COVID-19 pandemic in Germany was further highlighted in a survey carried out within the German oncology workforce by Trommer et al. in 2023 [22]. In this study, women also felt significantly more burdened during the pandemic. In academia, caring for children or others is also often considered to be a reason why women struggle to reach higher academic positions [31, 32]. Our study also showed that women in medical physics and biology are involved in care work at a younger age than men ((26–30 years in women vs. 31–35 years in men), Supplementary Fig. 1). This finding is in accordance with the general population in Germany, i.e. in Germany, women have their first child at an age of 31.7 and men at an age of 34.7 years, according to data from the Federal Statistical Office in 2023 [33]. In contrast, the age for first-time-mothers in academic women in Germany was 32 years in 2020, while 50% of women in academia were childless at an age of 35 years [34]. Of course, UCW or caring responsibilities are not the only potential driver of gender inequalities. A recent survey conducted by the ESMO Women for Oncology Committee highlights challenges women in the oncology workforce might face and emphasizes that gender inequalities in oncology careers can extend beyond family responsibilities [35]. While there has been a recent analysis of parity amongst physicians in the German radiation oncology field [36], this study was also limited to the medical workforce, while this survey included all major academic workgroups included in radiation research and radiation oncology. While the authors of this study would like to acknowledge that there are potential structural barriers for female career advancement, this exceeds the scope of the present work and was not content of the present survey.

Regarding fixed contracts, biologists are the subspeciality with the latest onset for permanent positions, as permanent positions appear in age groups 36–40 years for the first time. A trend that has also been observed elsewhere, as those that work mainly in research tend to have more temporary contracts than permanent positions in general [37]. In the biology group, it is especially noteworthy that almost twice as many male than female biologists were holding a permanent position (Fig. 2), while 72.9% of participating biologists are female (Supplementary Fig. 1). The gender discrepancy in research however, is a well-known problem in academia [38]. In general, among all subspecialities, participants involved in care work are holding a higher percentage of fixed contracts in contrast to all participants. This, on the other hand, could also be an age-dependent bias, as older participants are more involved in care work and older participants have a higher percentage of permanent positions (Fig. 2; Supplementary Fig. 1). Furthermore, within our cohort, especially in physician and biologist subgroups, males involved in care work were holding higher career positions than females involved in care work (Fig. 3). This trend is also visible in all participants, as 14% of senior physicians are male while only 10.8% of senior physicians are female, as well as for biologists where 18.8% of Group Leaders and 31.3% of Junior Group Leaders are male, while only 11.8 and 9.3% are female, respectively. Medical physicists on the other hand show a special characteristic, as more women involved in care work hold a full MPE than men involved in care work. When looking at the more classical career descriptions however, male and female medical physicists show more equal career outcomes than the other subspecialities (Fig. 3). However, as participation at this career level was rather low in the medical physicist group, no clear statements can be made here. Differences in career level between males and females are especially pronounced in biologists, as this is the cohort with the highest participation of females (72.9%), Supplementary Fig. 1.

The trends observed in our study are also well-known in other countries. Although the representation of women in radiation oncology faculty positions has increased over time, it still remains low compared to the number of men in this position. In 2019, only 29.1% of radiation oncology faculty were female [39]. Furthermore, women are also underrepresented in leadership positions in radiation oncology, e.g. as in 2021, only 17.4% of radiation oncology leadership positions were held by women in the US in 2021 [40]. More precisely, male radiation oncologists constitute for 86.1% of chairpersons and full professors compared to only 13.9% of female radiation oncologists [39, 41].

Some of the reasons for this are that women in radiation oncology, like many other specialties, face further obstacles such as perceived gendered societal norms resulting in them focusing more on teaching and clinical activities rather than research [40, 41]. This is also reflected in the results of our study, which showed that most participants were actively involved in teaching and supervision (57%). Still, especially those with care responsibilities showed a higher percentage in teaching and supervision (54% of the physicians, 76% of the biologists, 41% of the medical physicists, data not shown) compared to all participants [2]. This suggests that despite their unpaid care work, the young workforce takes on commitments and engages in their career that goes beyond daily routine work. It might also suggest that societal norms and expectations indeed lead to a higher involvement in this subarea of the whole spectrum of radiation oncology. Furthermore, a persistent gender pay gap, lack of funding, and administrative support for women, might lead to decreased professional confidence and career advancement [40, 41].

This is not only true in the medical field but also in biology and medical physics [13, 42, 43]. Statistical data of the winter term 2023/2024 in Germany has shown that 66.2% of biology students were female (57,351 biology students overall, 37,860 female biology students) [13, 42], while their numbers in leadership positions decline with increasing academic rank [43]. In prestigious biology laboratories led by male faculty members such as Nobel laureates, or investigators for prestigious institutes, a noticeable lack of female representation is found. For instance, within the labs of male Nobel laureates, male graduate students outnumber female graduate students by a ratio of 2:1, and male postdoctoral researchers outnumber female postdoctoral researchers by more than 3:1 [44]. In a study of medical biochemistry and genetics faculty in North America, more male faculty held higher academic ranks such as professor compared to the examined female faculty. Only 23% of professors were women, and only 11.6% were full professors [45]. In Germany, a similar trend can be seen as the percentage of women obtaining a PhD in academia in general is decreasing from 46.1 to 36.5% women undergoing habilitation and 28% women holding a full-time professorship [43]. This development is also represented in our data, as more male than female participants were holding permanent contracts and Group Leader positions. In medical physics, globally, only 28% of medical physicists are female according to a 2015 report by the International Organization of Medical Physics (IOMP) [46]. In the United States, only 23% of medical physicists are women, indicating a significant underrepresentation compared to other regions like Europe (34%) and Latin America (33%). With women holding only 12% of clinical leadership roles in medical physics in the US, 13.6% in Canada, and 18% in other countries combined [46]. This trend however, was not found in our study, as female medical physicists had more permanent positions and held a full MPE more frequently than their male colleagues.

There seems to be discrimination against employees involved in care work and also against women in their fertile years, as shown by studies that imply the time women tend to take off for childbirth and child care could be a potential reason for discrimination [47, 48]. In our study, we found no significant differences between the weekly working hours between employees with or without care work (Supplementary Fig. 2). Therefore, fear of reduced working hours in women during their fertile years seems to be unjustified.

We also found no significant influences of care work on research (Supplementary Fig. 3). There was a slight shift in favor of those carrying out care work not being involved in research anymore overall in the physics and medical subspecialities. This observation however, could be biased as care work is usually carried out in higher age groups that also tend to hold higher positions, as it can be seen in our study. Higher positions potentially go along with more responsibilities and lesser time for research. Another reason could be, that those that stated they do not carry out research, are not employed at a university, where there is a higher rate of research activity compared to other facilities. Our data thus suggests, that neither males nor females involved in care work in the field of radiation oncology or radiation research carry out less work in comparison to all participants. The majority of participants involved in care work further stated to make up for lost hours during the day in the evening (Fig. 4). While our data might suggest that there is a shift for research to be carried out more after regular working hours, a generalized fear of reduced working times for those involved in care work seems unjustified.

On the other hand, reduced possibilities and support especially for women leads to far-reaching consequences: while females represent over 50% of all medical school matriculants, their numbers decrease in higher positions [39–41, 49]. This does not only lead to an underrepresentation of women in higher positions, but also to persisting gender disparities in research productivity and authorships. Women radiation oncologists have fewer first-author publications, lower citation rates, and lower h‑indices compared to their male counterparts, even after controlling for factors like residency program size and advanced degrees. Furthermore, despite a rise in overall female authorship over the last decade, there remains a discouraging lack of progress in the representation of women in prominent authorship roles, such as first and last authors, in leading radiation oncology journals [41].

We also asked participants involved in care work, whether substitution rules are in place at their institution (Fig. 4). While for physicians involved in care work more male than female participants stated to always stay at home when their kids fell sick (m: 30.8%, f: 23.1%), in the biologist subgroup more female than male participants said the same (m: 0%, f: 50%). For medical physicists, numbers were equally distributed between male and female participants (m: 53.3%, f: 50%). While it is often thought that women with children show a higher number of absent days due to sickness of their children [50], a study in Sweden found that this is not the case, as women not involved in child care had even higher numbers of absent days due to sickness, and women with more than one child had similar sick days to women with one child [50]. This was especially true for women in higher skill levels occupations and managers. Here, sickness absence was increased in women with decreasing skill-level, especially in the years before childbirth in a study with 492,504 women [51]. Our study data therefore also backs up these findings. Most participants, both male and females, further state that part-time work is possible at their institution. However, numbers are highest in the medical physicist cohort. Likewise, medical physicists are also the subgroup with the highest amount of substitution rules, in comparison to physicians and biologists. As a lack of substitution rules has also been identified to be the biggest reason for a failure in part-time work in radiation oncology [52], introduction of clear substitution rules in all subspecialities might lead to better rates of successful part-time work.

A common assumption is, that a lack of compatibility of work and family or a lack of work life balance [53], is one of the main reasons for the young workforce to leave their jobs, also in radiation oncology and radiation research. In our cohort this was not the case (Fig. 5). Here, in the physician subgroup economic pressure was the biggest risk factors for terminating a career in radiation oncology, independent of gender, this is also true for physicians involved in care work. For biologists, uncertain contractual situations are the biggest risk factor for terminating a career in radiation research, independent of gender and also for females involved in care work. However, this perception shifts in males involved in care work. They identify a lack of future perspectives as biggest risk factor. For medical physicists, male participants identify economic pressure as biggest risk factor, female medical physicists are the only subgroup that does identify a lack of work/life balance as the biggest risk factor to leave radiation oncology. An alarming sign among the scientific landscape however, is the strong increase in fear of lack of future perspective, especially in male physicians and biologists involved in care work (physicians 53.8%; biologists 66.7%). Another problem is found in participants when asked if they feel sufficiently supported by their employer in their career plans (Fig. 6). In general, participants involved in care work feel less supported in their career goals than all participants. While for all participants, men in the medical field felt more supported than women by their employer regarding their career goals, this is reversed in participants involved in care work. Here, male physicians feel less supported than female physicians. One of the reasons why males involved in care work feel less supported and show a bigger fear of lack of future perspectives might be, that men involved in care work are less likely to ask for help [54] and thus do not receive sufficient support. Another potential reason is a lack of support due to perceived gender norms and expectations.

However, it is important to recognize that male academics engaged in unpaid care work encounter a distinct set of challenges, influenced by factors such as societal norms and gender expectations, which often associate caregiving responsibilities with women. The role of a caregiver often appears to conflict with traditional notions of masculinity for some men. Additionally, men involved in care work face workplace barriers and, like women, experience effects on their academic performance and career progression [55–58]. Indeed, a study on academic productivity in hematology showed that while both men and women face similar tasks related to caregiving, the effects differ significantly. Men involved in care work often experience a reduction in academic productivity due to their caregiving responsibilities, whereas productivity-levels in women remain largely unaffected. In the same study, participants involved in care work had fewer numbers of first- or senior-author publications. While male participants in general had more first- or senior-author publications and higher numbers of total publications than women, men with caregiving responsibilities significantly reduced all scientific outcomes in the examined cohort [59], suggesting a need for additional support.

In summary, there is an increasing need for recruitment of the young workforce into the field of radiation oncology and radiation research. Prejudices affecting hiring of women in their fertile years [48], especially in higher positions, counteracts these efforts not only directly, but also indirectly. While the representation of women in radiation oncology faculty positions has increased over time but still remains low, urgent measures need to be taken to counteract this [49]. The underrepresentation of women in higher-ranking faculty positions and leadership roles perpetuates a scarcity of female mentors and role models, which can further hinder the recruitment and retention of women in the field. This mentorship gap has been identified as a significant barrier to women’s career advancement into academic leadership roles in medicine [40, 41]. Data from our study indicate that existing caveats about hiring women in a certain age group are unjustified as no significant decrease in work hours or research was found. In our study, more males stated to be involved in care work, which brings along a unique set of challenges. While female participants involved in care work seemed to receive more support, males often felt a lack of future perspective and insufficient support in their career goals. This also potentially leads to a reduction in (scientific) output [59], although these effects were not visible in our cohort. This stresses the need for solutions and support for all workers, male and females, in radiation oncology and radiation research involved in care work, especially during the sensitive period with (very) young children, in order to avoid the loss of young parents in the field of radiation oncology and radiation research in the early stages of a career. Possible solutions could be the implementation of clear substitution rules, as it is shown in the medical physicist cohort.

## Conclusion

In order to retain highly qualified professionals in the field of radiation oncology and radiation research, we need to balance caring responsibilities and overcome gender inequality and professional barriers. This will ensure future prospects and sustainable career development, allowing us to provide the best patient care.

## Limitations

The survey was distributed at a conference, social media and by e‑mail to various representatives, thus we cannot be sure how many young academics were reached. Likewise, number of participants was limited in analyzed subgroups and we cannot control the answers given (e.g. in the numbers of hours worked per week, which partially seem to be rather high). Furthermore, we asked participants “Do you have children or relatives to be cared for”, but we did not expand this question in terms of percentage or hours the actual care work comprises and if there is a supportive partner or family, kindergarten or babysitter/nanny in the background. Thus, we could not really assess the actual burden of the paid work and unpaid work of the participants.

## Supplementary Information

**Supplementary Fig. 1: Gender distribution in the subspecialities as well breakdown of age distribution of participants involved in care work. **Data shows the general distribution of male, female, diverse, and non-disclosed participants in the three subspecialties. Next to the gender distribution within the subspecialties, an overview of the age distribution for all participants is given. Additionally, the age distribution for male and female participants involved in care work is shown in %.

**Supplementary Fig. 2:** **How many hours per week do you usually work in total? **We asked participants to tell us how many hours per week in total they work in average. Graphs for all participants and those involved in care work are plotted separately. Data is shown in [%].

**Supplementary Fig. 3: Do you carry out research within your regular working hours?** We asked participants to indicate whether they are able to carry out research work during regular working hours always or most of the time, or if they never carry out research during normal working hours. Graphs for all participants and those involved in care work are plotted separately. Data is shown in [%].

## Data Availability

The data presented in this survey are available from the corresponding author upon reasonable request in an anonymized manner.
