# Supplementary figures and images for: Balancing barriers: Family, career, and gender equality in radiation oncology and radiation research—An interdisciplinary prospective survey among the young workforce
Source: Strahlenther Onkol. 2025 May 27;202(6):602–18. doi: 10.1007/s00066-025-02402-2 (PMC13216192; doi:10.1007/s00066-025-02402-2)

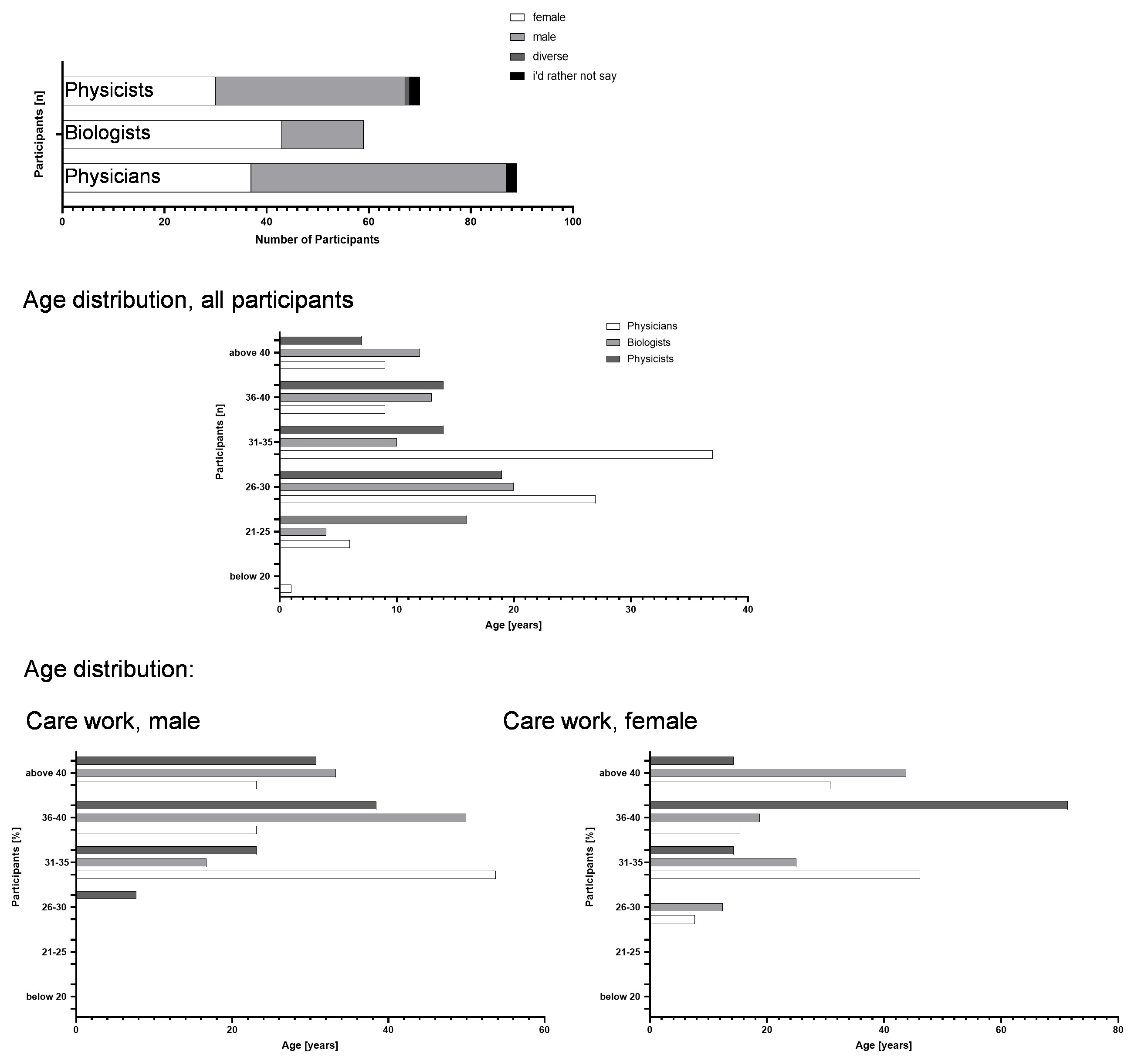

Supplement: Supplementary file 1 — Supplementary Fig. 1: Gender distribution in the subspecialities as well breakdown of age distribution of participants involved in care work. Data shows the general distribution of male, female, diverse, and non-disclosed participants in the three subspecialties. Next to the gender distribution within the subspecialties, an overview of the age distribution for all participants is given. Additionally, the age distribution for male and female participants involved in care work is shown in %. [file 66_2025_2402_MOESM1_ESM.jpg]

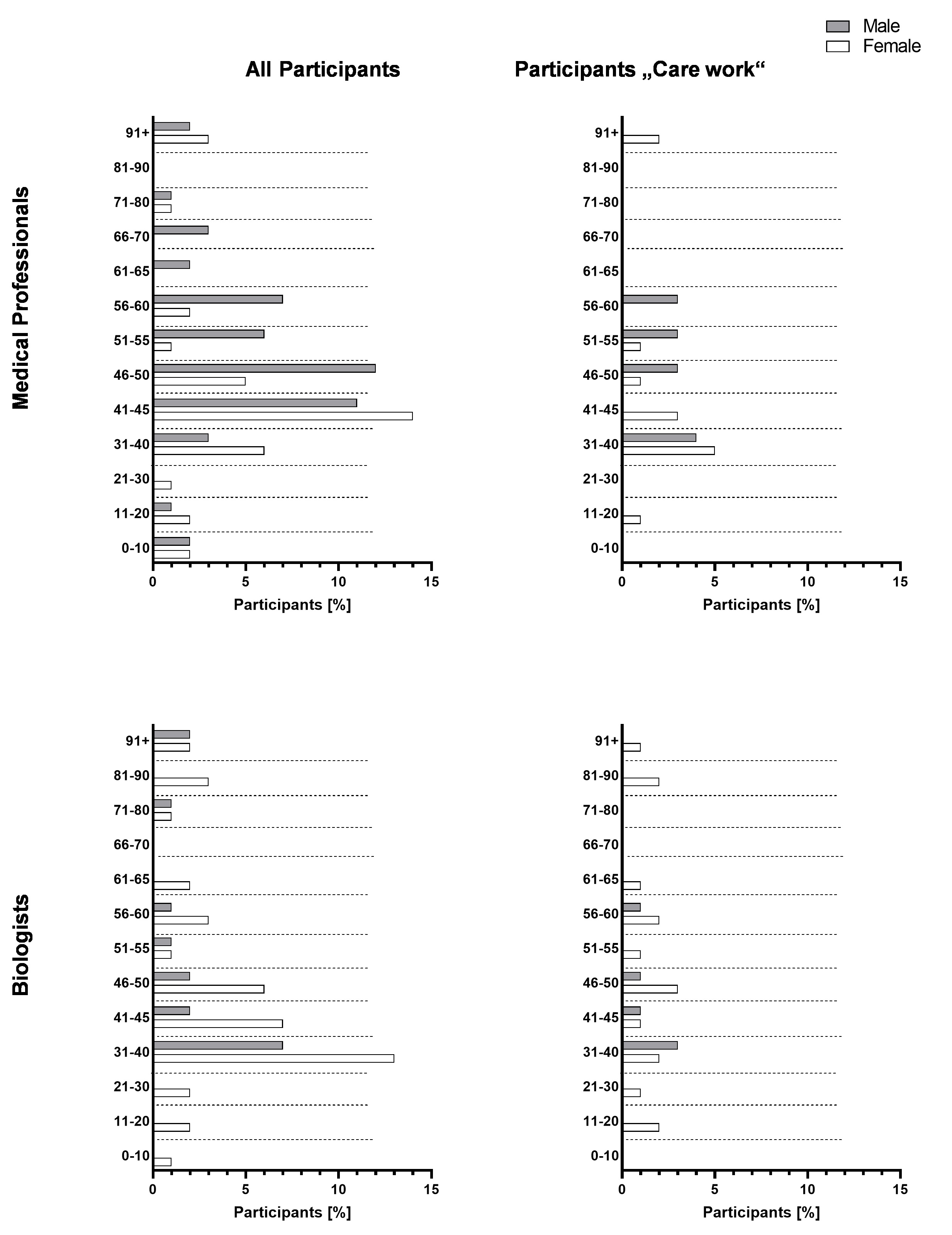

Supplement: Supplementary file 2 — Supplementary Fig. 2: How many hours per week do you usually work in total? We asked participants to tell us how many hours per week in total they work in average. Graphs for all participants and those involved in care work are plotted separately. Data is shown in [%]. [file 66_2025_2402_MOESM2_ESM.jpg]

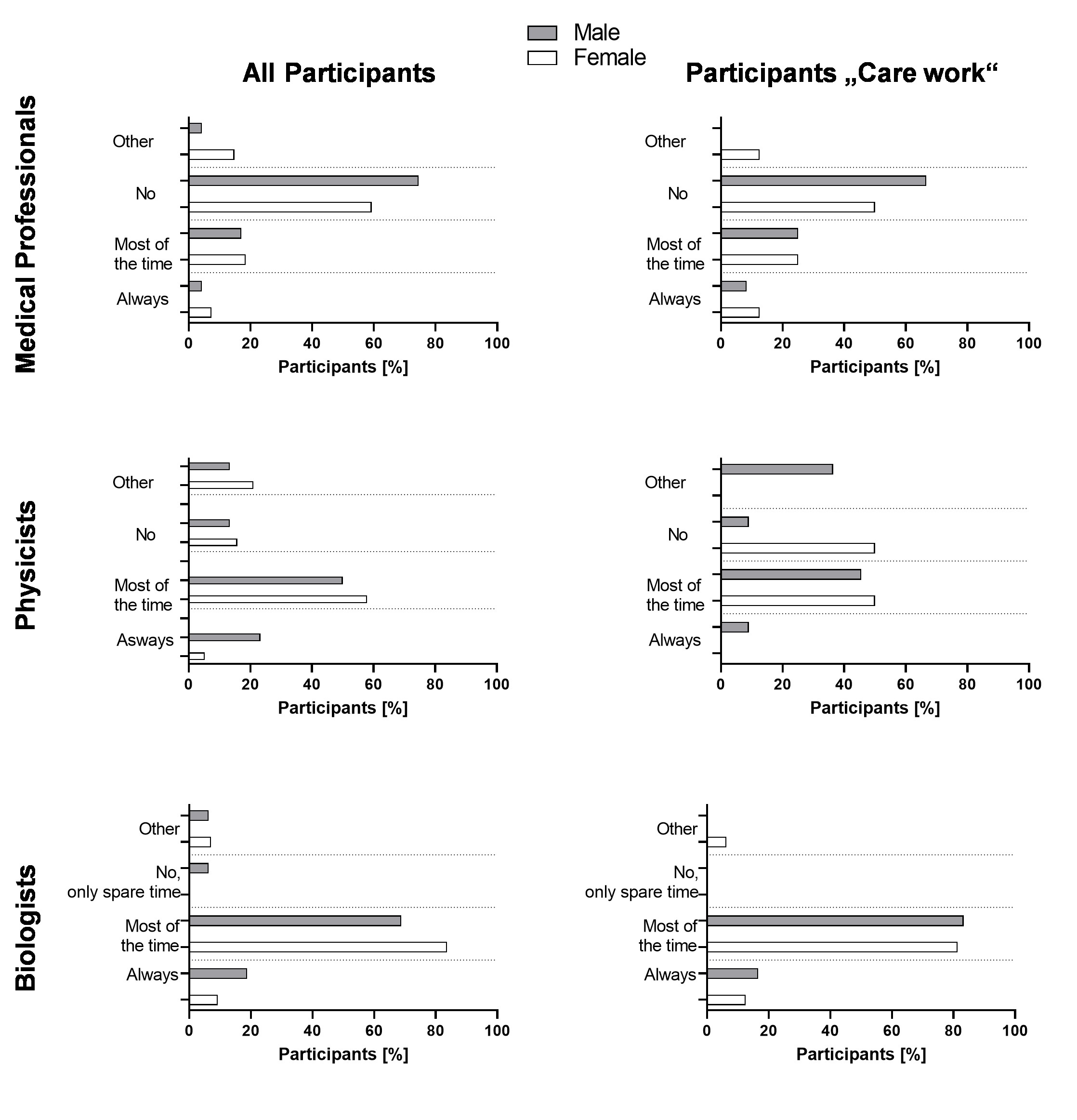

Supplement: Supplementary file 3 — Supplementary Fig. 3: Do you carry out research within your regular working hours? We asked participants to indicate whether they are able to carry out research work during regular working hours always or most of the time, or if they never carry out research during normal working hours. Graphs for all participants and those involved in care work are plotted separately. Data is shown in [%]. [file 66_2025_2402_MOESM3_ESM.jpg]
